# Supplementary material for: K-Ras Binds Calmodulin-Related Centrin1 with Potential Implications for K-Ras Driven Cancer Cell Stemness
Source: Cancers (Basel). 2023 Jun 7;15(12):3087. doi: 10.3390/cancers15123087 (PMC10296094; doi:10.3390/cancers15123087)
Supplement: Supplementary file 1 [file cancers-15-03087-s001.zip › cancers-2407877-supplementary.pdf]

Supporting Information

K-Ras Binds Calmodulin-Related Centrin1 with Potential Implications for K-Ras Driven Cancer Cell Stemness

Ganesh babu Manoharan, Christina Laurini, Sara Bottone, Nesrine Ben Fredj and Daniel Kwaku Abankwa *

Cancer Cell Biology and Drug Discovery Group, Department of Life Sciences and Medicine, University of Luxembourg, L-4362 Esch-sur-Alzette, Luxembourg

* Correspondence: daniel.abankwa@uni.lu

Supplementary Figures

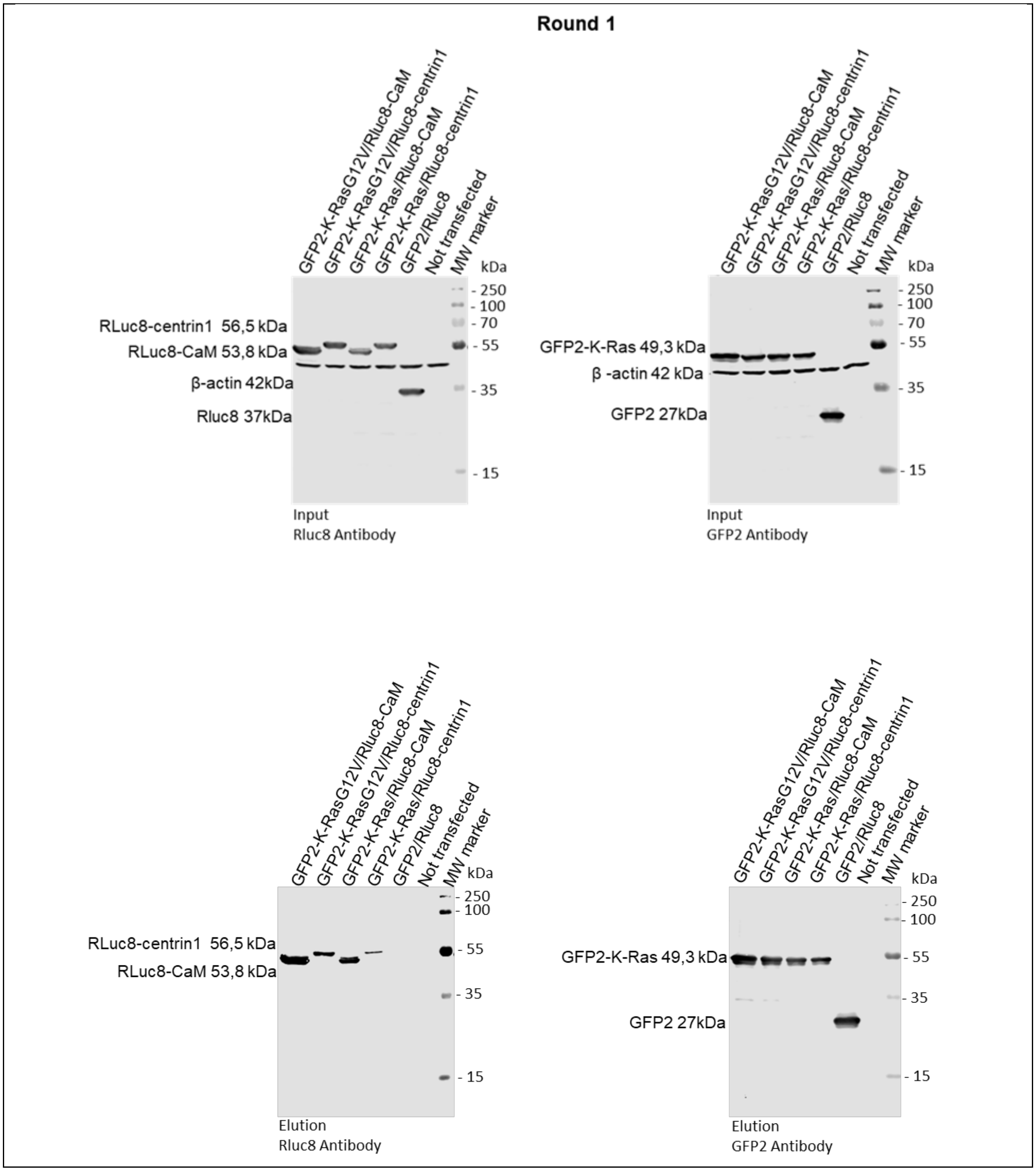

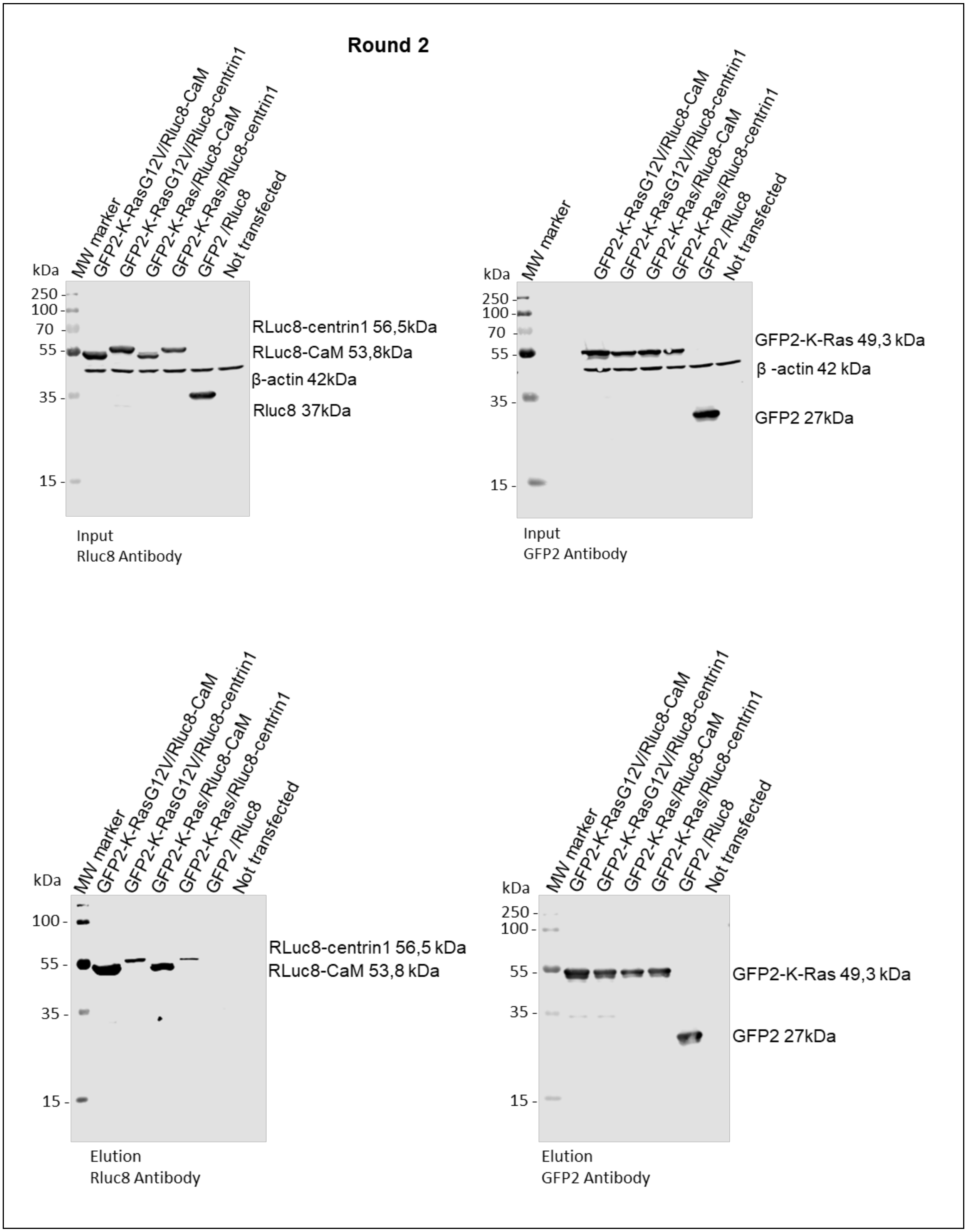
Round 2
MW marker
GFP2-K-RasG12V/Rluc8-CaM
GFP2-K-RasG12V/Rluc8-centrin1
GFP2-K-Ras/Rluc8-CaM
GFP2-K-Ras/Rluc8-centrin1
GFP2 /Rluc8
Not transfected
kDa
250
100
70
55
35
15
RLuc8-centrin1 56,5kDa
RLuc8-CaM 53,8kDa
β-actin 42kDa
Rluc8 37kDa
Input
Rluc8 Antibody
GFP2-K-Ras 49,3 kDa
β -actin 42 kDa
GFP2 27kDa
Input
GFP2 Antibody
RLuc8-centrin1 56,5 kDa
RLuc8-CaM 53,8 kDa
Elution
Rluc8 Antibody
GFP2-K-Ras 49,3 kDa
GFP2 27kDa
Elution
GFP2 Antibody

Round 3

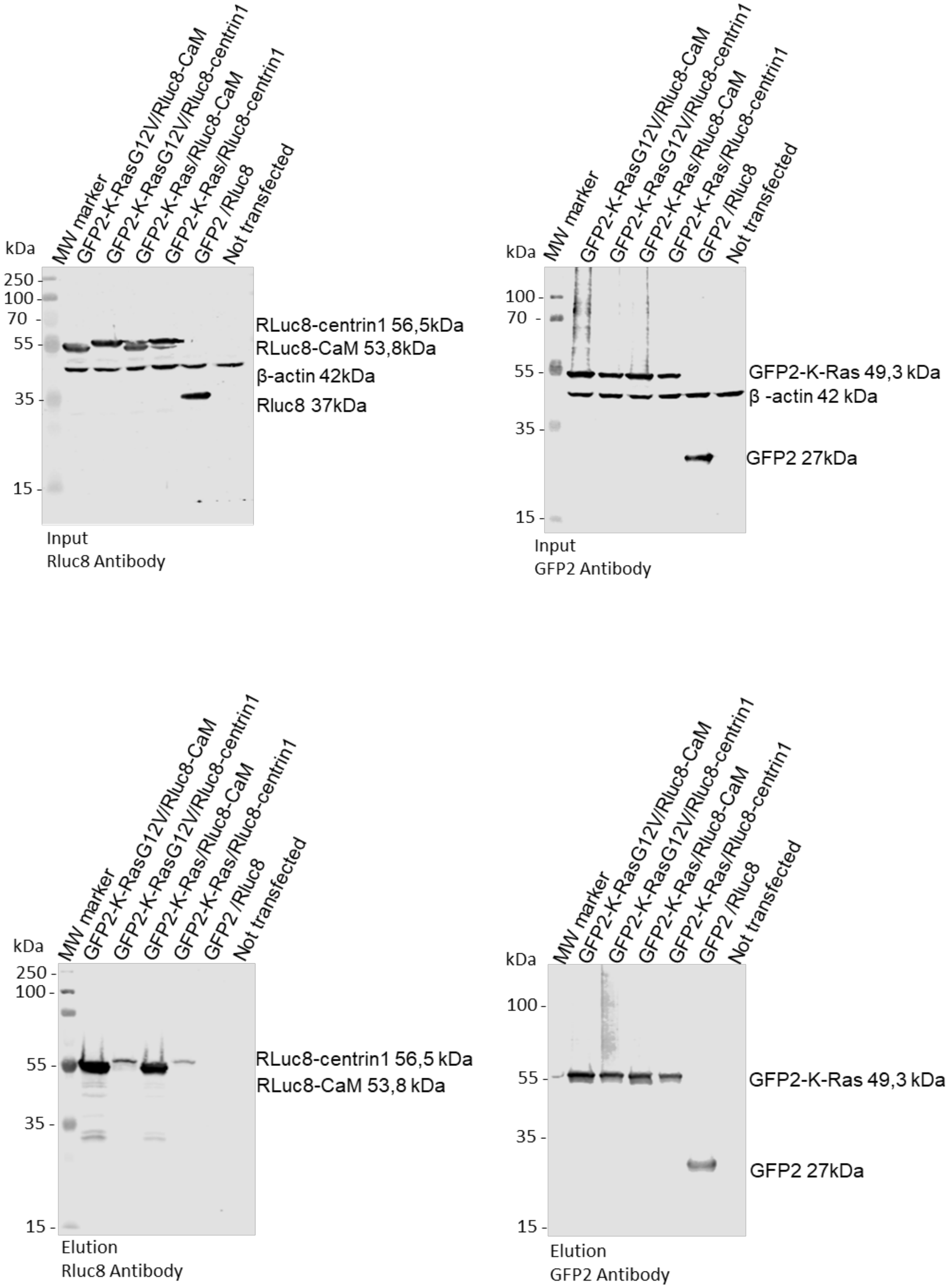
MW marker
GFP2-K-RasG12V/Rluc8-CaM
GFP2-K-RasG12V/Rluc8-centrin1
GFP2-K-Ras/Rluc8-CaM
GFP2-K-Ras/Rluc8-centrin1
GFP2 /Rluc8
Not transfected
kDa
250
100
70
55
35
15
RLuc8-centrin1 56,5kDa
RLuc8-CaM 53,8kDa
β-actin 42kDa
Rluc8 37kDa
Input
Rluc8 Antibody
GFP2-K-Ras 49,3 kDa
β -actin 42 kDa
GFP2 27kDa
Input
GFP2 Antibody
RLuc8-centrin1 56,5 kDa
RLuc8-CaM 53,8 kDa
Elution
Rluc8 Antibody
GFP2-K-Ras 49,3 kDa
GFP2 27kDa
Elution
GFP2 Antibody

Figure S1 (Supporting data for Figure 2 A,B). Co-immunoprecipitation of Rluc8-CaM or Rluc8-centrin1 with GFP2-K-RasG12V or GFP2-K-Ras. Full blots of 3 biological repeats (Round 1, 2 and 3) showing co-immunoprecipitation data from lysates of HEK293-ebna cells expressing combinations of Rluc8-/ GFP2-tagged fusion proteins: GFP2-K-RasG12V/ Rluc8-CaM, GFP2-K-RasG12V/ Rluc8-centrin1, GFP2-K-Ras/ Rluc8-CaM, GFP2-K-Ras/ Rluc8-centrin1. And as a control the tags GFP2/ Rluc8 alone were tested. Cropped blot from round 1 is shown in **Figure 2A.** Quantification from these 3 biological repeats is shown in **Figure 2B.**

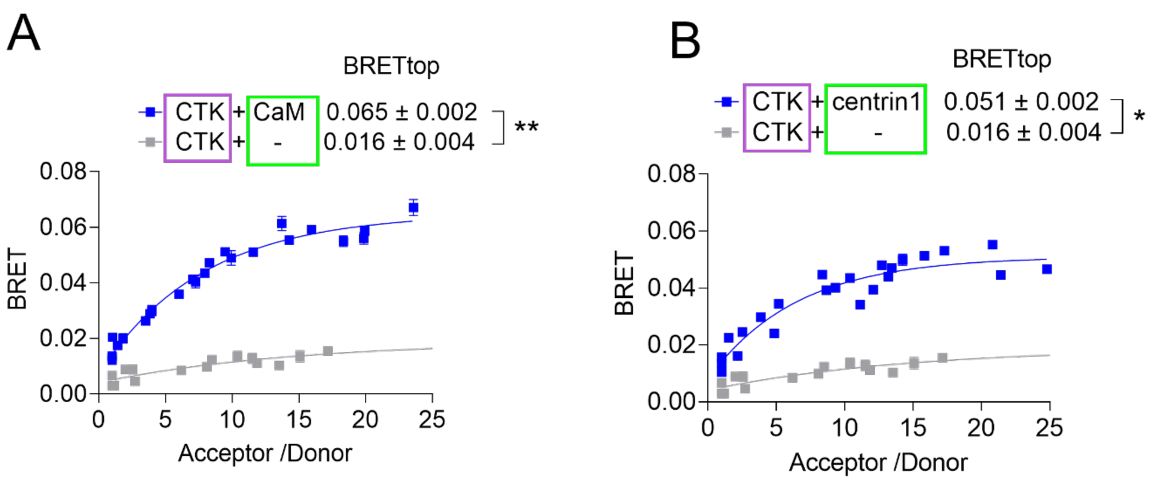

Figure S2 (Supporting data for Figure 2 C,D). Control BRET donor saturation titration curves showing significantly lower BRET than of the weakest interacting BRET-pair. The background BRET resulting from the titration of Rluc8-CTK and GFP2 alone is given in grey squares. For comparison, the BRET-curves of the weakest studied interaction of CTK with CaM or centrin1 is shown. The BRET donor protein is boxed purple, acceptor protein is boxed green. Data from 2 – 3 biological repeats are plotted together and the statistical analysis was performed using F-test. Note that the BRETtop values are slightly lower than the values reported in **Figure**

4 C,D for CTK/ CaM and CTK/ centrin1 interactions, due to variations in the methods. Here, the amount of donor plasmid used for titration was 20 ng and no DMSO treatment was done. For better comparison of the background BRET, the x-axis, [Acceptor] /[Donor] ratio is normalised using RFU/RLU of the data point where equal amounts of donor and acceptor plasmids were transfected.

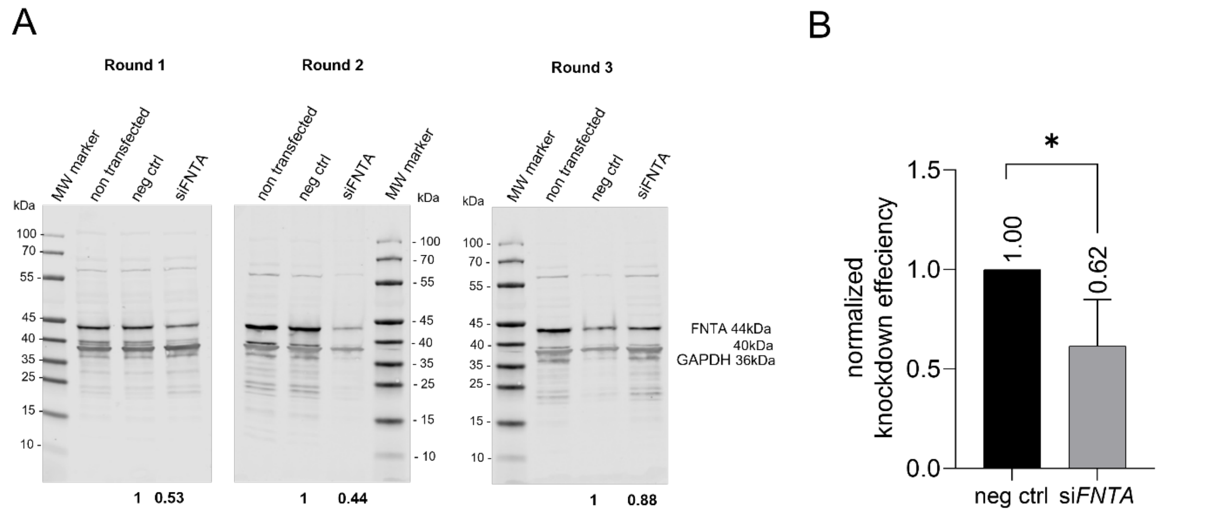

Figure S 3 (Supporting data for Figure 5C). Validation of knockdown of *FNTA* in HEK293-ebna cells. (**A**) Full blots showing the knockdown of si*FNTA* in HEK293-ebna cells from 3 biological repeats. Numbers below each lane indicate the FNTA-protein (shared α-subunit of farnesyl- and geranylgeranyl-transferases) band signal normalised to the GAPDH expression. Cropped blot from round 2 is shown in **Figure 5C**. Non-transfected lane corresponds to cells treated with transfection reagent and neg ctrl lane corresponds to negative control siRNA transfected cells. (**B**) Statistical analysis was performed using one sample t-test.

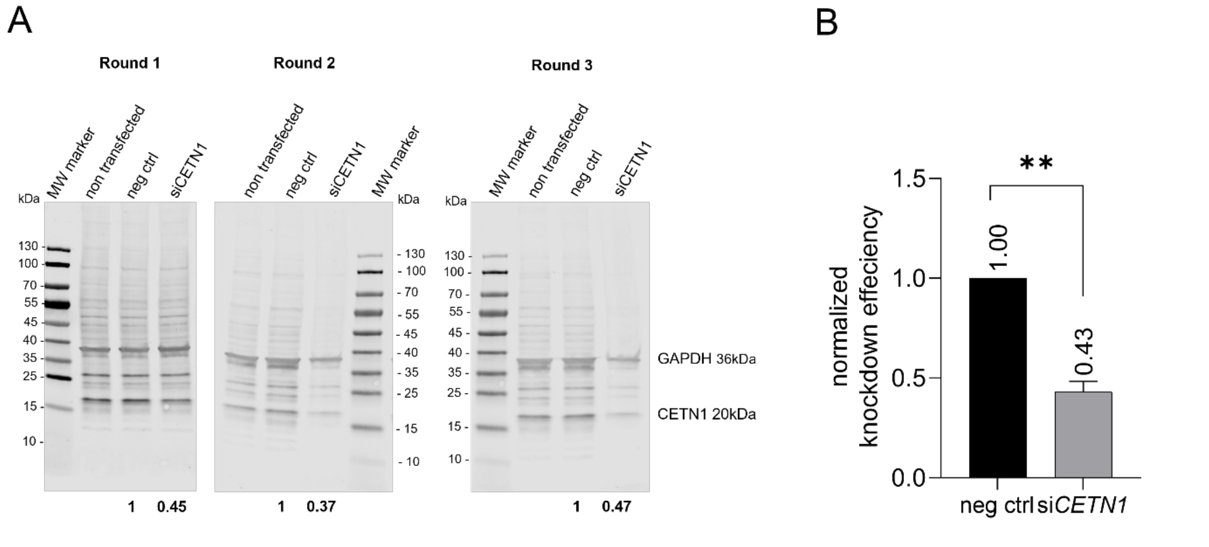

Figure S 4 (Supporting data for Figure 5D). Validation of knockdown of *CETN1* in HEK293-ebna cells. (**A**) Full blots showing the knockdown of si*CETN1* in HEK293-ebna cells from 3 biological repeats are presented. Numbers below each lane indicate the centrin1 (CETN1) protein band signal normalised to the GAPDH expression. Cropped blot from round 2 is shown in **Figure 5D**. Non-transfected lane corresponds to cells treated with transfection reagent and neg ctrl lane corresponds to negative control siRNA transfected cells. (**B**) Statistical analysis was performed using one sample t-test.

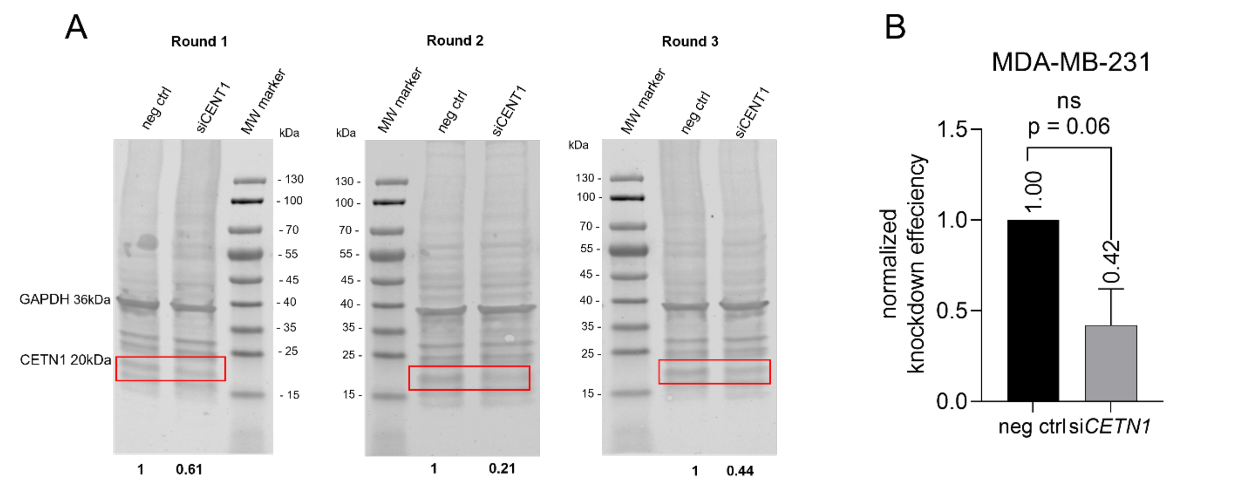

Figure S5 (Supporting data for Figure 6A). Validation of knockdown of *CETN1* in MDA-MB-231 cells. (**A**) Full blots showing the knockdown of si*CETN1* in MDA-MB-231 cells from 3 biological repeats. Numbers below each lane indicate the centrin1 protein band signal normalised to the GAPDH expression. A red box is drawn around the centrin1 (CETN1) expression range on the blots. Neg ctrl lane corresponds to negative control siRNA transfected cells. (**B**) Statistical analysis was performed using one sample t-test.

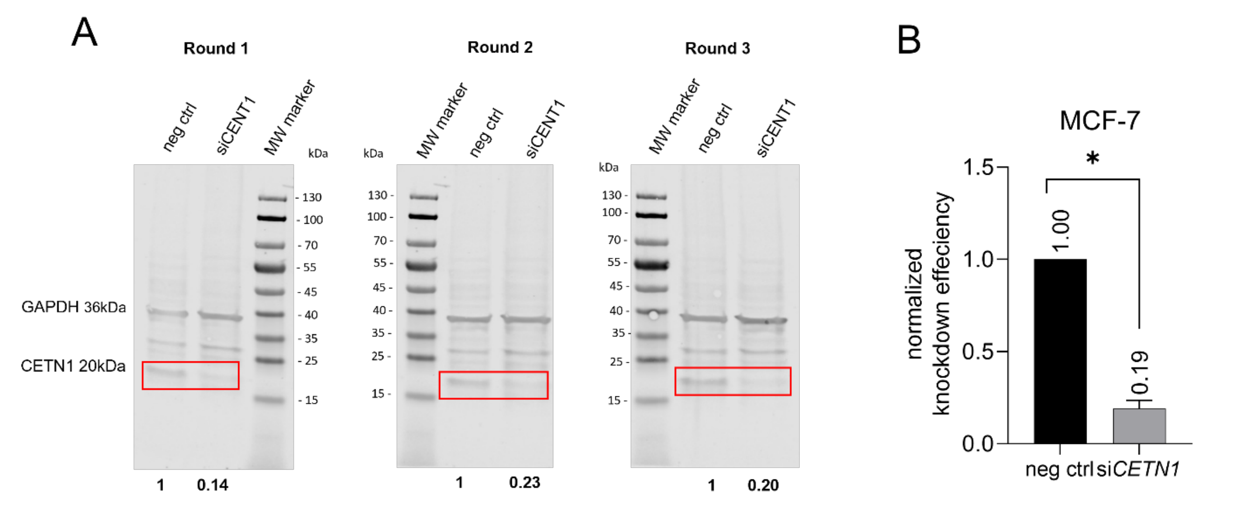

Figure S6 (Supporting data for Figure 6B). Validation of knockdown of *CETN1* in MCF-7 cells.

(**A**) Full blots showing the knockdown of si*CETN1* in MCF-7 cells from 3 biological repeats. Numbers below each lane indicate the centrin1 protein band signal normalised to the GAPDH expression. A red box is drawn around the centrin1 expression range on the blots. Neg ctrl lane corresponds to non-targeting pool siRNA transfected cells. (**B**) Statistical analysis was performed using one sample t-test.
